# Collaborations and Networks Within Communities for Improved Utilization of Primary Healthcare Centers: On the Road to Universal Health Coverage

**DOI:** 10.3389/ijph.2024.1606810

**Published:** 2024-06-03

**Authors:** Chinelo Obi, Iheomimichineke Ojiakor, Enyi Etiaba, Obinna Onwujekwe

**Affiliations:** ^1^ Health Policy Research Group, College of Medicine, University of Nigeria, Enugu Campus, Enugu, Nigeria; ^2^ Department of Health Administration and Management, Faculty of Health Science and Technology, University of Nigeria, Enugu, Nigeria

**Keywords:** community actors, health service accessibility, primary healthcare, social support, Universal Health Coverage

## Abstract

**Objectives:**

Community involvement depends on the level of linked and targeted activities for health by community members. This study examines the collaborations employed within communities to ensure sustainable access and improved use of healthcare in the community.

**Methods:**

This study was conducted in rural and urban local government areas in Anambra, Kano, and Akwa-Ibom, Nigeria. About 90 in-depth interviews and 12 focus group discussions were conducted with community stakeholders and service users. The findings were transcribed and coded via thematic analysis, guided by the Expanded Health Systems framework.

**Results:**

Various horizontal collaborations in communities foster increased use of PHC services; promoting community health. Major horizontal collaborations in these communities were community-led, primary health facility-led, and Individual-led collaborations. Their actions revolved around advocacy, building and renovating PHC centers, equipping facilities, and sensitization to educate community members on the need to utilize services at PHC centers.

**Conclusion:**

Strategic involvements and collaborations of local actors within communities give rise to improvements in the utilization of primary healthcare centres, reportedly resulting in improved access to PHC healthcare services for community members.

## Introduction

The Primary healthcare (PHC) system is an effective, efficient, and equitable approach to enhancing health and achieving Universal Health Coverage (UHC) [1]. The Astana declaration on primary healthcare reaffirmed the primal place of PHC and its key pillars including community participation, in strengthening health system for the achievement of UHC. Redirecting efforts to strengthen health systems towards a primary healthcare approach is essential for driving meaningful and effective change [2].

Community involvement is a crucial pillar in supporting primary healthcare. It entails engaging community members in promoting their own health and wellbeing, as well as that of their families and the community at large. This participation extends to collaborations on strategies that address the healthcare needs of community members [3]. Furthermore, community members also have the opportunity to exercise their right to make decisions that influence their health. This active involvement of community members not only enhances the effectiveness of primary healthcare services but also empowers individuals to take control of their own health [4].

In some contexts, high levels of community involvement in health-related activities, have led to a conceptualization of community health systems (CHS) being defined as *“. . .the set of local actors, relationships, and processes engaged in producing, advocating for, and supporting health in communities and households outside of, but existing in relationship to, formal health structures”* [5]. It has also been conceptualized as the ‘grey zone’ between the public health system, non-governmental organizations (NGO) and private health system [6], comprising community actors that transcend beyond Community Health Workers and include community groups, informal health providers, faith organizations, sporting groups, social networks, and non-government sectors such as housing, education, and social development that support close to community providers that contribute to healthcare in the community [7]. However, in Nigeria, whilst there are no defined CHS boundaries, the term is sometimes used interchangeably with the PHC system [8] there is a clear recognition of community involvement in health-related activities and health services, hence the need for collaboration with the formal PHC system.

Collaboration by the different stakeholders at the community level is important to ensure that the CHS works optimally, whether independently or as part of the PHC system. Collaboration is the merging of activities and knowledge, necessitating a partnership characterized by shared authority and responsibilities. It involves coordination of members to achieve shared goals; cooperation of team members by respecting each other’s opinion; shared decision making, which relies on open communication, trust, and power balance; partnerships where members work together in equity [9].

Access to healthcare and improvement in the health and health equity of the populace can be influenced by collaborations and endeavors of various organizations from national and local governments, schools, agencies, and community organizations [10, 11]. This influence comes from the impact that living environment, policies, and economic conditions have on the health of the population [12]. Partnerships and collaborations beyond the health field have been proposed as a way to ensure improved health [13].

In the global space, cross-country collaborations have been used to address public health concerns like tobacco use [14]. There have also been established collaborations within countries in diverse contexts. They exist as public service collaborations and community alliances in a particular state or interstate arrangements. In the United Kingdom and the United States, inter-sector partnerships are used among policymakers to address health concerns by using healthcare organizations and non-healthcare organizations to coordinate healthcare services and other existing social services to improve the health of the public [11, 15]. Collaborations can sometimes comprise a smaller populace in a town, local government, or a village [16]. Duties in the coalition could be voluntary or mandatory to achieve a common course [13].

Collaborations could be affected by management and financial issues, as well as cultural and accountability barriers. When reviewed, even established partnerships may not be as resilient or prepared to enhance community health as their reputation would imply [17, 18]. However, several studies have pointed out key traits of successful collaboration as mutual trust, effective communication, balanced structures/roles, and aligned goals among the members of the associated organizations and their leaders [19, 20]. Other factors that led to effective collaboration were shared hosting, team meetings, evaluation of partnership, and previous history of successful partnership [19, 20].

Increased access to healthcare services can be fostered through collaborations. Equitable access was seen in the less privileged population and people accessing mental healthcare [21, 22]. Some studies reported a reduction in access as the outcome of a failed plan of collaborations [13].

However, poor access to healthcare services, especially to PHC services is the major reason behind preventable deaths in Sub-Saharan Africa [23]. Barriers to accessing the use of PHC services are mostly systemic challenges like lack of coordination and defective healthcare workforce [24]. Heightened trust in traditional medicine can also hinder the use of primary healthcare facilities. Cultural barrier is another major hindrance [25]. Other barriers include unavailability of infrastructure, lack of functional equipment, lack of manpower, absenteeism among healthcare workers, misinformation, long waiting times, attitude of healthcare workers, and financial hindrances [26, 27].

Many of the constraining factors are traced back to poor levels of community involvement and participation in the development and implementation of strategies for improving health within their communities. Some women have to seek permission before seeking care, which is a restriction to timely care. In Northern Nigeria, women in Purdah cannot seek healthcare even when in labour, unless the husband gives his permission or accompanies her to the hospital [26].

Collaborations and networking that exist in communities as part of community involvement and participation in the PHC system remain largely underexplored. Active community and engagement have been reported in different settings, with positive contributions to community health.

This paper contributes to the literature on the role of collaborations and networking among actors within the community, and how they can contribute to making the PHC system more effective for all. It investigates the benefits, challenges, and strategies for effective collaborations and networking, and provides practical recommendations for stakeholders. It is hoped that the evidence that the paper provides will benefit healthcare professionals and policymakers by providing insights to inform decision-making processes, thereby strengthening community wellbeing.

### Conceptual Framework

**FIGURE 1:**
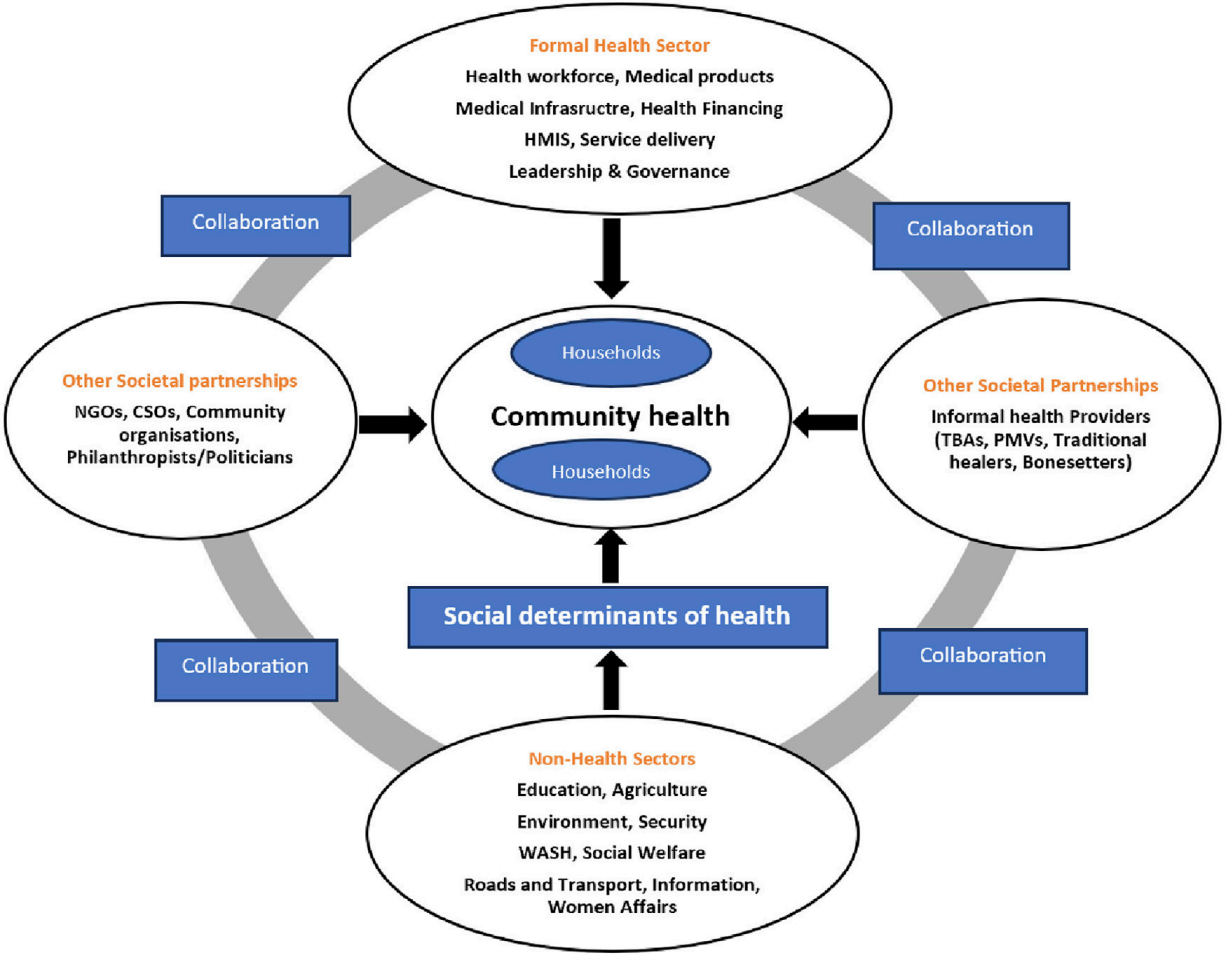
The Expanded Health System building blocks, adapted from Sacks, et al (Global, 2018).

The conceptual framework for the study was adapted from the expanded health system framework proposed by Sacks et al in 2019 [11], which recognizes “societal partnerships” between the formal PHC system building blocks and community actors including informal providers, and community organizations (Figure 1). We focused on how community participation and partnerships/collaborations improved coverage and increased access to the utilization of PHCs. The expanded health system framework recognizes the inclusion of community action, household provision of health and partnerships with other non-health sectors, and a multiplicity of stakeholders [11].

The framework shows different potential combinations of collaborations between the non-health sector and other societal partnerships (community groups and informal health providers), to directly contribute to community health or indirectly through one or more social determinants of health. In our study, there is an independent focus on collaboration with the non-health sectors, and collaboration with other societal partnerships.

## Methods

### Study Design and Setting

The study adopted a qualitative cross-sectional study design to explore insights into community participation and involvement in community healthcare. The method was chosen to describe the phenomena from the participants’ perspective. This study was reported according to the consolidated criteria for reporting qualitative studies. See Table 1 for details. Three states from three of the six geopolitical zones in Nigeria were selected for the study. The states selected were Kano (North-west), Akwa-Ibom (South-south), and Anambra (South-east). In each state, two local government areas (LGAs) were selected, with one predominantly rural setting and the other urban.

**TABLE 1:** Summary of the study using the consolidated criteria for reporting qualitative studies (Nigeria, 2021–2022).

	Description	Remarks	Page no
Domain 1: Research team and reflexivity			
*a*) *Personal Characteristics*			
1. Interviewer	Which author/s conducted the interview or focus group?	OO and EE led, trained, and guided the interview and focus group. Two authors (CO and IO) conducted the in-depth interview and the focus group discussion	—
2. Credentials	What were the researcher’s credentials?	OO: PhD	—
		EE: PhD	
		CO: MSc	
		IO: MSc	
3. Occupation	What was their occupation at the time of the study?	OO: Physician with director of research responsibilities	—
		EE: Academic	
		CO: Academic	
		IO: Academic	
4. Gender	Was the researcher male or female?	OO: Male	—
		EE: Female	
		CO: Female	
		IO: Female	
5. Experience and training	What experience or training did the researcher have?	OO and EE are experienced researchers in qualitative studies and have collectively published numerous qualitative research articles	—
		CO and IO attended a workshop on “how to conduct qualitative research”	
*b*) *Relationship with participants*			
6. Relationship established	Was a relationship established prior to study commencement?	During the mobilization of the participants solely for this study	—
7. Participants’ knowledge of the interviewer	What did the participants know about the researcher? *e.g*., personal goals, reasons for doing the research	The participants did not know any of the researchers. Though they all knew that the interview was for research purposes	—
8. Interviewer characteristics	What characteristics were reported about the interviewer/facilitator? *e.g*., Bias, assumptions, reasons and interests in the research topic	The characteristics of each author have been reported above	—
Domain 2: Study Design			
a. Theoretical framework			
9. Methodological orientation and theory	What methodological orientation was stated to underpin the study?	Thematic analysis	5
*Participant selection*			
10. Sampling	How were participants selected? e.g., purposive, convenience, consecutive, snowball	Purposive sampling was used to select participants	4
11. Method of approach	How were participants approached? *e.g*., face-to-face, telephone, mail, email	Participants were recruited face-to-face	4
12. Sample size	How many participants were in the study?	A total of 102 participants were in the study	4
13. Non-participation	How many people refused to participate or dropped out? Reasons?	None	4
c. *Setting*			
14. Setting of data collection	Where was the data collected?	Data were collected in offices, health facilities, and homes, usually based on the participants’ preference	5
15. Presence of non-participants	Was anyone present besides the participants and researchers?	No. Just the participants and the researchers	5
16. Description of sample	What are the important characteristics of the sample?	The sample comprised of formal healthcare providers, informal healthcare providers, religious bodies, community leaders and community members	4
d. Data collection			
17. Interview guide	Were questions, prompts, and guides provided by the authors? Was it pilot-tested?	Interview guides were provided by researchers involved in the study and they were pilot-tested. Adjustments were made to the interview guides after the pilot	5
18. Repeat interviews	Were repeat interviews carried out? If yes, how many?	There were no repeat of interviews	—
19. Audio/visual recording	Did the research use audio or visual recording to collect the data?	Audio recording was used for data collection	5
20. Field notes	Were field notes made during and/or after the interview or focus group?	Field notes were taken during the interview and focus group discussion. They were used to assist in the analysis of the transcribed audio recordings	5
21. Duration	What was the duration of the interviews or focus group?	The interview and FGD lasted about an hour	5
22. Data saturation	Was data saturation discussed?	It was not	
23. Transcripts returned	Were transcripts returned to participants for comment and/or correction?	There were no return of transcripts	5
Domain 3: Analysis and findings			
*a*) *Data analysis*			
24. Number of data coders	How many data coders coded the data?	A total of 12 trained researchers worked in pairs	5
25. Description of the coding tree	Did authors provide a description of the coding tree?	No, it was not provided	—
26. Derivation of themes	Were themes identified in advance or derived from the data	The expanded health system framework was identified in advance and used in coding	5
27. Software	What software, if applicable, was used to manage the data?	Excel was used for data management	—
28. Participant checking	Did participants provide feedback on the findings?	Participants did not provide any feedback on the findings	5
*b*) *Reporting*			
29. Quotations presented	Were participant quotations presented to illustrate the themes/findings?	Yes, quotations were presented and identified to illustrate the themes using participants’ role and the city	5–9
	Was each quotation identified? *e.g*., participant number		
30. Data and findings consistency	Was there consistency between the data presented and the findings?	There was consistency between the data and the findings	5–9
31. Clarity of themes	Were major themes clearly presented in the findings?	Major themes are clearly presented	5–9
32. Clarity of minor themes	Is there a description of diverse cases or a discussion of minor themes?	No, there were no descriptions of the diverse cases or discussion of minor themes presented	—

### Study Participants Selection

Participants were purposively selected based on their roles and involvement in health service provision to ensure a representative sample. They included policymakers, health programme managers, formal healthcare providers, informal healthcare providers, Civil Society Organizations (CSOs)/Non-Governmental Organizations (NGOs), community leaders, and community groups, to ensure diversity in views. The participants were recruited through a face-to-face encounter, where they agreed to partake in the study. At the end of the interview process, 102 successful interviews were recorded, comprising 90 in-depth interviews (IDIs) and 12 focus group (FGD) discussions with male and female groups of respondents, as summarized in Table 2.

**TABLE 2:** Summary of respondents and interviews by state (Nigeria, 2021–2022).

Respondent category	Akwa Ibom	Anambra	Kano
Health sectorpolicymakers	3	2	3
Health program managers	2	2	1
Formal healthcare providers	4	3	4
Informal healthcare providers	13	7	11
Intermediary health workers	—	—	3
Private health sector	—	4	—
CSO/NGO	2	4	3
Community or Religious leader	7	5	7
(FGD) Community groups/Service users (Women)	2	2	2
(FGD)Community groups/Service sers (Men)	2	2	2
*Total (Males)*	*18*	*18*	*25*
*Total (Females)*	*17*	*13*	*11*
*Total per state*	*35*	*31*	*36*

### Data Collection

Data was collected using pre-tested separate interview guides for the IDIs and FGDs respectively. The interview guides focused on questions that elicited responses on how healthcare is organized and operated within communities as well as the level of community involvement in health. Interviews were conducted face-to-face and were audio-recorded to avoid missing any details during transcription. Field notes were also made during the interview to assist in the analysis of the transcribed recordings. Informed consent was collected from each of the participants after informing them of the scope and purpose of the study and their right to participate voluntarily. Permission was taken before audio-recording and the interview lasted for approximately 1 hour. Interviews were undertaken in offices, health facilities, and homes, usually based on the participants’ preferences. English and Igbo were used for interviews in Anambra state; Hausa and English were used in Kano state. In Akwa Ibom state, only English was used to administer the interview.

### Data Analysis

English interviews were transcribed verbatim while the Hausa, Efik and Igbo interviews were translated into English Language and then transcribed. The transcripts were not returned to participants for comments or corrections. Thematic analysis was incorporated, with broad themes focusing on community organization and participation, multi-sectoral collaboration, and partnerships developed from the extended health systems building framework. Transcripts were used to develop the codebook and the codebook was subsequently refined through repeated meetings to improve the quality. Relevant narratives were placed under suitable themes. Transcripts were coded twice by two different researchers to check accuracy.

## Results

Major actors within the community include formal healthcare providers, informal health providers, community organizations, community leaders, and community members. Each of these actors played different roles that contributed to enhancing the use of primary healthcare facilities in communities as summarized in Table 3.

**TABLE 3:** Actors involved in community collaborations (Nigeria, 2021–2022).

Position of actors in the health system	Actors
Internal actors in the health system	Health workers in the PHC
	Health Facility Committee
	Ward Development Committee
External actors in the health system	Informal healthcare providers: TBAs, herbalists, bonesetters and PMVs
	Religious bodies
	Youth organizations
	Community Individuals

**FIGURE 2:**
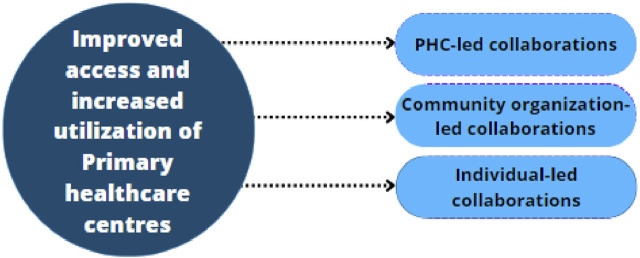
Identified informal horizontal collaborations contributing to increased utilization of Primary Healthcare Facilities (Nigeria, 2021–2022).

The roles they played centered around sensitization/awareness creation, advocacy, and paying for impoverished community members to access care. They also donated lands for building of PHCs, renovated PHCs, and created structures that will promote the penetration of health programmes and interventions. These roles played by various community members to improve access to health facilities are presented under the collaborations that occurred in the community: PHC-led collaboration, Community organization-led collaboration, and Individual-led collaboration. These are depicted in Figure 2 and described below.

### PHC-Led Collaboration

Health workers in the PHC facilities and informal providers like the traditional birth attendants (TBAs), Patent Medicine vendors (PMVs), bonesetters, and herbalists collaborated to promote health in the community. The health workers’ major engagement strategy was creating awareness through health education. The health workers in the communities refreshed the skills of informal health providers, thereby boosting their knowledge of referrals and the need for referrals. This was considered imperative since a majority of these communities visit the informal providers to provide care to them.

*The TBAs are doing well. It’s still difficult to convince everybody to come out to the facility. So, we meet with them, refresh their skills, and visit them. When we bought the consumables, we took some to them and begged them to send these women to come in for antenatal and go through the antenatal processes at least. We negotiate with them for antenatal care because the people trust them more than they trust us *(Officer-in-Charge (OIC), Akwa Ibom)*.*

Informal providers began collaborating with the formal providers by helping them organize their clientele to be receptive and accept formal health interventions when referred.

*. . .we’re enjoying the training because the training is encouraging us, and unlike before, most of the women now go to PHC facilities after giving birth at home. We influenced it because of the training we received. We pass the information we receive to them. Giving birth at home is equally rare now. There are times when I do not have to take the delivery but instead take the pregnant woman to the hospital *(TBA, Kano)*.*

Another narrative. . .

*In some cases, if a wounded patient comes to me for treatment, I send him to the hospital first to have the wound treated, and then when the wound is healed, I will work on the bone from the injured area *(Bonesetter, Akwa Ibom)*.*

### Community Organization-Led Collaboration

Healthcare workers from the PHCs collaborate with the village heads, ward heads, Imams, and other community groups, like influential people within the community, who can influence the community members to access services in the PHC facilities. These organizations like the Ward Development Committee (WDC) and Health Facility Committee (HFC) facilitate the use of primary healthcare facilities by creating awareness in the community. These Organisations have also been deliberate in setting up community structures such as the co-groups and the Voluntary Community Mobilizers (VCM) in northern Nigeria to bring about the necessary community presence needed for the penetration of health programmes and interventions.

*The WDC as well as the facility health committee create awareness by sensitizing the community. Moving from door to door to enlighten pregnant women on the importance of antenatal care is one of the jobs of the women in the organization, the men on the other hand, educate their gender in the mosque or any gathering. The men’s education is centered on the importance of men taking their family members to the hospital at early stage of illness and to refrain from total reliance on traditional medicines (WDC treasurer, Kano). A farmer in Akwa-Ibom reiterated the same point.*

*Yes, these organizations have tried a lot. They have tried on the issue of pregnant women, they have done a lot, they educate them. Some people who come there, are educated and told what to do. Recently, they have organized seminars, educating community members on the need to use the PHC. They also educate them on some ailments, their prevention and cure* (Community leader, Anambra state).

Youth organisations assisted the community by footing the bills of impoverished patients, and by taking part in sanitation. Community youths also spearhead advocacy. Youth leaders in Akwa Ibom advocated for efficient and qualified health workers for their health facilities to heighten usage. The government honoured their request promptly. They do this by themselves or partner with others.

*The community youth help in checking blood pressure and sugar level during outreaches, but if it involves the eyes, they partner with Niger Optical. Medical personnel from Niger Optical take charge of eye checkups *(CSO, Anambra).

Churches and mosques give their congregation opportunities to use the PHC facilities in the communities by collaborating mostly with the PHC facilities. In some scenarios, they refer some of their members for free drugs/care and also coordinate activities in PHCs. In the same light, the school system educates students on the benefits of utilizing PHC facilities, which creates the needed awareness.

*We are currently running a program that registers eligible patients to receive free drugs in the hospital. We were invited to coordinate the process of net distribution, sweeping, and cleaning of the hospital. We work with the nurse in charge of the hospital; she supports us a lot. There are many incidences where she lent her vehicle to us for us to complete our task* (Imam, Kano).

*In the mosque or the church, the church leaders tell their members to visit the healthcare facilities in the case of an illness or an outbreak* (Chairman Farmers Association, Kano).

Religious leaders also follow up on pregnant women and ensure they receive care.

*I personally follow up on pregnant women for prenatal care, ante-natal care, and post-natal care. So, I always refer them to either the PHC facility or to the general hospital, as the case may be* (*Clergy, Anambra*).

### Individual-Led Collaboration

Individuals in the community donate plots of land for erecting health facilities, and collaborate to sponsor building or renovations of health facilities.

*I donated that plot of land where the health centre is erected. This was over 19 years ago. Nothing in this world is bigger than land *(Village head, Akwa-Ibom)*.*

Another narrative was also reported.

*One of the rich men in our community built this building some years back. We conduct antenatal and immunization there. Some years back, the community members went to seek funds for the facility. They explained that they wanted an additional ward in the hospital so he agreed and built it *(Health attendant, Kano)*.*

The elite members of the communities also pay for the less privileged ones to boost the use of the facilities.

*From time-to-time individuals pay bills for those who cannot afford to. A few years ago, there was a community member who paid up bills accumulated by pregnant teenagers. Okay, he did it for one or 2 years and stopped. At times other individuals will come in, and pay up bills for those who cannot pay their hospital bills *(Medical Doctor, Igboukwu)*.*

There were also narratives of health schemes introduced by a community elder to ensure that everyone gets the treatment they need. This scheme enabled the healthy ones to finance the health of the sick ones. In Igboukwu Anambra state there was a reported case where the community members paid 100 naira premium monthly dues to access health. This way, healthy individuals pay for those who require care.

They also solicit help to build, renovate, and buy equipment for the health facilities to enhance usage.

*We usually solicit help from affluent persons in society, we do not ask for money rather, we put forward requests to supply medications, renovate or build new wards in the hospital and so on. Those drugs and injections are used to treat poor patients. The bags of cement here were contributed by a person who was born and raised in this hospital quarters, he is the son of the former medical director, we solicited his help to renovate a ward in the hospital but he opted to build a whole new pediatric ward for us. We also sort hospital bills for some patients and also clean and disinfect the facility* (Deputy OIC in Kano).

The health providers in the PHCs also invest in the PHC by equipping the facility when necessary. They do this using their personal funds.

*We equipped the labour room built by the chairman with the money that we get from the service account. The money paid for the minor casualty service we are rendering was what we used in the equipment of the labour room* (CHEW and OIC, Kano).

### Perceived Influences of Collaborations on Community Health

Various collaborations in communities that foster the use of PHC facilities is promoting health, fixing misconceptions, bringing PHC facilities closer to the people, and improving services in these facilities.

An extract reported the benefits of the proximity of PHCs.

*Things have really improved unlike when healthcare facilities were far, but today they are more accessible. When the government took over this place after we erected it, we were not happy, but today they are doing things we would not have done if we had been the one managing it. A lot has changed and so far, we are pleased* (Business woman, Anambra).

Misconceptions are being phased out by sensitizations and continuous awareness creation.

*In the olden days most of the people did not come because they heard that most of the children come to the facility, take routine immunizations and get killed. But, through community mobilizations and sensitization, they’re aware that all these are wrong* (CHEW and OIC, Kano).

Improved use of PHCs was also noted.

*There are lots of improvements when it comes to pregnant women. Women now come for regular check-ups once they find out that they are pregnant. We send them for laboratory tests and take care of them as the case may be. Women who gave birth at home also come to the hospital with these babies for check-ups and immunization* (women, Kano).

## Discussion

The findings show that actors in all study areas were keen on ensuring that health works for all. Collaboration and participation were seen among community individuals and organizations to ensure improvement in the use of PHC facilities. Community members at the level of religious bodies, primary healthcare facilities, and informal providers collaborate and strategize to enhance the use of PHC facilities. Community committees and philanthropists in the organization also contribute to the enhancement. This collaborates with a Mexico study which reported that patients barely had access to advanced treatment, before the involvement of the community in the activities of the PHCs in rural Mexico [28]. It also aligns with studies done in Cross River state and Ibadan, Nigeria. They reported how the involvement of community members worked towards ensuring that quality care is available for all. They sensitized, created awareness, and mobilized resources to ensure the use of available healthcare services [29, 30]. On the contrary, Gholipour et al., 2023 reported community involvement as theory rather than practice in Iran. This could stem from not setting it up as an independent service unit [31].

Collaborations with local actors took different approaches. Training and retraining of informal providers contributed invariably. It enhanced the minds of these providers and opened their minds to the possibility of better healthcare service delivery for community members. This worked as much as involving them in healthcare services. A study conducted in Uganda reported that TBAs tried to bridge the gaps of power dynamics in homes because of the trust that some men have in them. This arose when women sought care in the informal sector because their husbands did not permit formal healthcare services [32]. This study reported that men are free when interacting with TBAs concerning their wives’ problems during pregnancy and childbirth. This creates an avenue for these TBAs to step in and give them reasons why their wives need to access care informal healthcare centres [32].

Sensitizing community members on the need to enroll and utilize the PHC facilities was another collaboration measure. This collaborates an Indian study that buttressed community involvement in healthcare delivery through the use of participatory learning action. It emphasized the need for the mechanism to be included through professionalism and teamwork attitude to bring about the necessary transformation in the health system [33]. A Belgium study maintained that increasing health literacy of a communities can optimize the use of healthcare services [34].

An equipped hospital with keen staff is an avenue to increase the people’s trust and ensure their reliability on the system, to enable utilization. Brals revealed that an upgraded healthcare facility tends to increase the utilization of healthcare services [35]. A study in Indonesia reported that one of the major factors to improve quality of care is by having high-quality medical staff, which in turn increases the use of health facilities. These staff should keep getting trained to enhance efficiency [36]. A study in sub-Saharan Africa reported that health workers were kept on their toes by monitoring absenteeism, the quality of healthcare delivered, and the expenditures in the system. Health workers in some sub-Saharan Africa need enlightenment on the need for community involvement in healthcare [4]. A similar study in South Africa reported that committees monitored health workers’ absenteeism and quality of healthcare delivery. However, these committees were considered illegitimate because they lacked transparency, and participation in selecting group members were male-dominated. These committee members were barely involved in the planning process, owing to health workers considering them uneducated and uninformed [37].

Enabling the proximity of health centers to the people occurred through collaborations. Most of these communities contributed to building the health centre by donating land, soliciting support, and combining resources to build and restructure hospitals. Cost minimization for community members who cannot afford healthcare was eminent too. Resources were mobilized and secured and used to ensure that those that need healthcare but cannot afford it, could access it. Collaborations and strategies also had community members making transport arrangements to enable access. This aligns with a Cross River study. They reported that community members were involved in building health centres and engaged in many developmental activities to better the lives of those in the community. The finding agrees with Adie et al., 2014 that activities are effective with proper mobilization [29].

The involvement of religious leaders was an approach that enhanced the use of PHC facilities because some communities consider their opinion final and the words of God. Their collaboration with formal providers contributed to a change of heart of certain men and women in the community. Akinloye’s study reported that religious leaders are seen as change ambassadors in Nigeria. He pointed out that this is the avenue for motivating and training them to advocate, educate and train their members on the need to utilize the primary healthcare centres in the community. A move that could do justice more in the development and the sustenance of PHC centres than the media [38].

Evidence from this research showed that these collaborations and varying involvements resulted in improved use of primary healthcare facilities. The result is an improvement in the health of community members. Maternal deaths and under-five mortalities were at bare. The finding from this study corroborates a study done in Korea which showed increased community involvement and capacity to enhance the community health status [39]. This also aligns with an Australian study that confirmed that involvements and engagements with community members were attributed to healthcare quality, access, utilization, and responsiveness, which in turn results in improved health outcomes in Australia [40].

A major limitation of the study is that it purely looked at horizontal collaborations within the community without involving vertical collaborations which involved the government. Future studies can include vertical collaborations that occur in communities.

Strategic involvements and collaborations of actors within communities give rise to improved access to PHC services for community members resulting in increased utilization of primary healthcare centres, and subsequent improvement in the health of the community members. Governments at all levels, especially at the local government level should formalize and strengthen the present informal community involvement in health by systematically incorporating the involvement for strategic strengthening of the PHC system for achieving UHC.
